# Relationships Between Fiber in Feedlot Diets and Growth Performance of Beef Cattle

**DOI:** 10.3390/ani15223266

**Published:** 2025-11-11

**Authors:** Michael L. Galyean

**Affiliations:** Department of Veterinary Sciences, Texas Tech University, Lubbock, TX 79409-2123, USA; michael.galyean@ttu.edu

**Keywords:** beef cattle, byproduct feeds, feedlot, growth performance, neutral detergent fiber, roughage

## Abstract

A database (110 treatment means from 23 studies) was derived from the peer-reviewed literature to evaluate the effects of various measures of dietary fiber on growth performance of feedlot cattle. The measures of diet fibrousness were the percentage of traditional roughage sources in the diet, the percentage of neutral detergent fiber (NDF) supplied by traditional roughages, and the total dietary concentration of NDF. The growth performance variables evaluated were dry matter intake, average daily gain, gain–feed ratio, and hot carcass weight. Mixed-model regression methods were used to adjust for random slope and intercept effects associated with studies, and study-adjusted data were evaluated to assess the effects of each of the measures of fibrousness on each of the measures of growth performance. The quality of the regression relationships was assessed by determining the significance of slope and intercept estimates, the root mean square error and the associated coefficient of variation, and the coefficient of determination (r^2^). In contrast to a previous analysis of literature data, 60% of the studies in the present database included fibrous byproducts (primarily wet corn gluten feed and wet or dry distillers grains plus solubles). Among the growth performance variables, measures of fibrousness were most highly correlated with dry matter intake (total consumed per day or percentage of average body weight), and among the measures of fibrousness, total dietary NDF concentration consistently accounted for the largest percentage of variation in growth performance variables. For dry matter intake as a percentage of body weight, total dietary NDF concentration accounted for 63.3% of the variation in dry matter intake, compared with 19.6% and 29.9% for NDF from roughage and percentage of roughage in the diet, respectively. Similarly, total dietary NDF concentration accounted for 58.1% of the variation in total dry matter consumed per day, compared with NDF from roughage (18.3%) and the dietary percentage of roughage (25.9%). For each 1% increase in total dietary NDF, dry matter intake increased by approximately 0.023% of body weight or 0.11 kg/d. Average daily gain and hot carcass weight were not strongly associated with measures of fibrousness (r^2^ values ranged from 0.001 to 0.122), reflecting the ability of feedlot cattle to compensate for lower dietary energy concentrations as NDF increased in the diet, at least within the ranges of NDF evaluated in the current database. As expected, with increased dry matter intake and little change in average daily gain, the gain–feed ratio was negatively associated with measures of fibrousness, with an r^2^ value of 0.296 for the total dietary NDF concentration. Although more research is needed on the role of physically effective NDF in feedlot diets, present data confirm previous observations about the relationship between the total dietary NDF concentration and dry matter intake, extending this relationship to feedlot diets that contain substantial proportions of fibrous byproducts. The total dietary NDF concentration in feedlot diets should be an effective basis for exchanging dietary ingredients to maintain a similar growth performance of feedlot cattle.

## Simple Summary

Sources of fiber are vital to the diets of feedlot beef cattle. Traditional roughages like hay and silage are often a major source of fiber in feedlot diets in the United States, but the inclusion of fibrous byproducts has increased significantly over the past two decades. A database was created from the peer-reviewed literature to assess the relationships between three measures of dietary fibrousness (percentage of traditional roughage sources in the diet, percentage of neutral detergent fiber supplied by traditional roughages, and total dietary concentration of neutral detergent fiber) and growth performance (dry matter intake, average daily gain, gain–feed ratio, and hot carcass weight). Using regression methods to adjust for random effects associated with the 23 studies in the database, total dietary neutral detergent fiber concentration was found to be strongly related to dry matter intake, with intake increasing by 0.023% of average body weight or 0.11 kg/d for each 1% increase in total dietary neutral detergent fiber concentration. Average daily gain and hot carcass weight were not strongly related to measures of fibrousness, but gain–feed ratio was negatively associated with total dietary neutral detergent fiber concentration. Feedlot producers should be able to achieve stable growth performance by using dietary neutral detergent fiber concentration as a basis for exchanging dietary ingredients, particularly traditional roughages and fibrous byproducts.

## 1. Introduction

Fiber has long been recognized as a vital part of ruminant diets. Indeed, in most ruminant production systems, fibrous components supply a significant proportion of the dietary energy. In feedlot beef production, however, dietary fiber sources are minimized to maximize the inclusion of grains and other concentrate feeds. For example, in a survey of feedlot nutritionists in the United States [1], traditional roughage sources (hay, silage, and high-fiber byproducts like cottonseed hulls and corn stalks) ranged from 6 to 12% of the diet. In recent years, digestible fiber sources from grain milling (e.g., wet and dry corn distillers grains and wet corn gluten feed) have increased substantially in feedlot diets, with inclusion rates from 10 to 30% of the dietary dry matter (DM) being common [1].

Traditional roughage sources, although included at a low percentage in feedlot diets, play important roles in ruminal fermentation and digestion, and ultimately growth performance. Compared with all-concentrate diets, adding a small percentage of roughage to feedlot diets increases intake of DM and thereby maximizes intake of net energy [2]. Moreover, roughage addition potentially affects ruminal pH, digesta kinetics [2,3], and the health of ruminal and perhaps intestinal tissues, leading to relationships with metabolic disorders like rumenitis and liver abscesses [4].

Despite the widely recognized effects of dietary roughage in feedlot diets, relationships between the inclusion of fibrous ingredients and the growth performance of feedlot cattle have not been studied extensively. To define the relationship between DM intake (DMI) and roughage inclusion in feedlot diets, Galyean and Defoor [2] compiled and analyzed literature data from feedlot studies in which roughage concentrations and sources were varied. Their results indicated that dietary neutral detergent fiber (NDF) supplied by roughage was more highly related to DMI as a percentage of body weight (BW) than the dietary percentage of roughage, leading the authors to conclude that equalizing the NDF supplied by roughage should be an acceptable basis for exchanging roughage sources in feedlot diets. In a subsequent analysis of the data from Galyean and Defoor [2], the relationship of the total dietary NDF concentration to DMI was also considered [5], with the suggestion that it also would be an effective tool for exchanging roughage sources.

To the author’s knowledge, a comprehensive analysis of the relationships between the percentage of dietary roughage, NDF supplied by roughage, and total dietary NDF concentrations and feedlot growth performance responses other than DMI has not been conducted. Thus, the objective of this current work was to create a literature database that included not only DMI but also other growth performance measurements to examine these relationships in cattle fed diets typical of those currently used by the feedlot industry.

## 2. Materials and Methods

### 2.1. Study Selection and Database Development

To reflect published data that were not included in the review of Galyean and Defoor [2], the peer-reviewed literature published between January 2004 and May 2025 was appraised using search engines (e.g., Google Scholar or specific journal websites) to identify studies with feedlot beef cattle that evaluated the effects of roughage source or concentration, and the related effects of NDF source and concentration, on growth performance. For inclusion in the database, studies were required to have growth performance data for cattle fed for an extended period until harvest and dietary composition data, particularly NDF, or at least the ability to calculate dietary components from dietary ingredients. Twenty-three studies (110 treatment means) were identified for inclusion in the database. Growth performance data included days on feed, initial and final BW, DMI, average daily gain (ADG), gain–feed ratio (G:F), and hot carcass weight (HCW). Dietary composition data included NDF, crude protein (CP), starch, and tabular concentrations [6] of net energy for maintenance and gain (NEm and NEg, respectively). All studies included data for CP, but values for dietary NDF and starch were not always reported. In such cases, diet composition information in the paper, along with tabular feed composition data [6], was used to calculate these values, resulting in 23% of the NDF values and 57.3% of the starch values being calculated from composition data. In addition to dietary composition data, the percentages of roughage and fibrous byproducts in the dietary dry matter (DM) were recorded, with percentages of NDF in these dietary components calculated from either reported or tabular NDF values depending on the availability of this information in the study. Descriptive statistics for the database are shown in Table 1, as well as the means and standard deviations for within-study ranges of measures related to dietary fiber. The complete database in spreadsheet format may be downloaded at https://www.depts.ttu.edu/afs/burnett_center/feedlot-fiber-database.php, accessed on 6 November 2025, and full citations for the studies included in the database are listed in Appendix A.

### 2.2. Statistical Analyses

An a priori decision was made to focus analyses on dietary roughage, NDF supplied by roughage, and total dietary NDF, all expressed as a percentage of the dietary DM, as these variables were the primary ones evaluated previously [2,5]. In addition, other feed composition variables (e.g., starch, CP, NEm, and NEg) that might be of interest were correlated to the measures of fiber, particularly NDF (r = −0.42, 0.26, −0.33, and −0.35 for starch, CP, NEm, and NEg, respectively; *p* < 0.006), suggesting the potential for problems with multicollinearity if included in multiple regression models along with measures of fibrousness.

The PROC MIXED of SAS (Version 9.4) was used to develop prediction equations for growth performance variables (DMI, % of BW and kg/d; ADG, G:F, and HCW) based on each of three independent variables (dietary roughage, NDF from roughage, and total dietary NDF). The models included random intercept effects with the “study” factor as the subject and an assumed unstructured covariance structure. Random slope effects were also included in all models; however, the model for ADG with the percentage of roughage in the dietary DM as the independent variable would not converge with random slope effects and, therefore, was fitted with only the random intercept effects. To scale the data to a common intercept, study-adjusted data [7] were created for each observation in the data set. Residual plots for study-adjusted data were evaluated to ensure normality of residuals.

The database included potential study weighting information, specifically the standard errors for the growth performance data reported in the publications and the number of observations per treatment. The mixed-model regressions were conducted both with and without weights for ADG, DMI, G:F, and HCW, where the weight was the inverse of the study variance, which was calculated from the standard error and number of observations per treatment. Ultimately, the decision was made to report the unweighted values. This decision reflected uncertainty regarding the reported standard error values in the various studies, many of which were randomized block designs. Thus, the differences in standard error values among studies could reflect block variance rather than lower precision of measurement. In addition, weighted analyses consistently had larger Akaike Information Criterion values than unweighted analyses, except for G:F, for which the values were approximately equal, suggesting a generally lower quality of model fit with weights included.

## 3. Results

**Figure 1:**
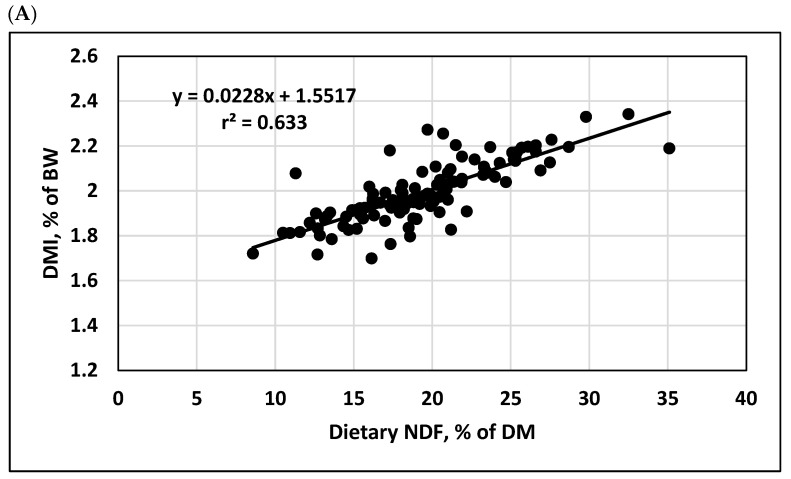

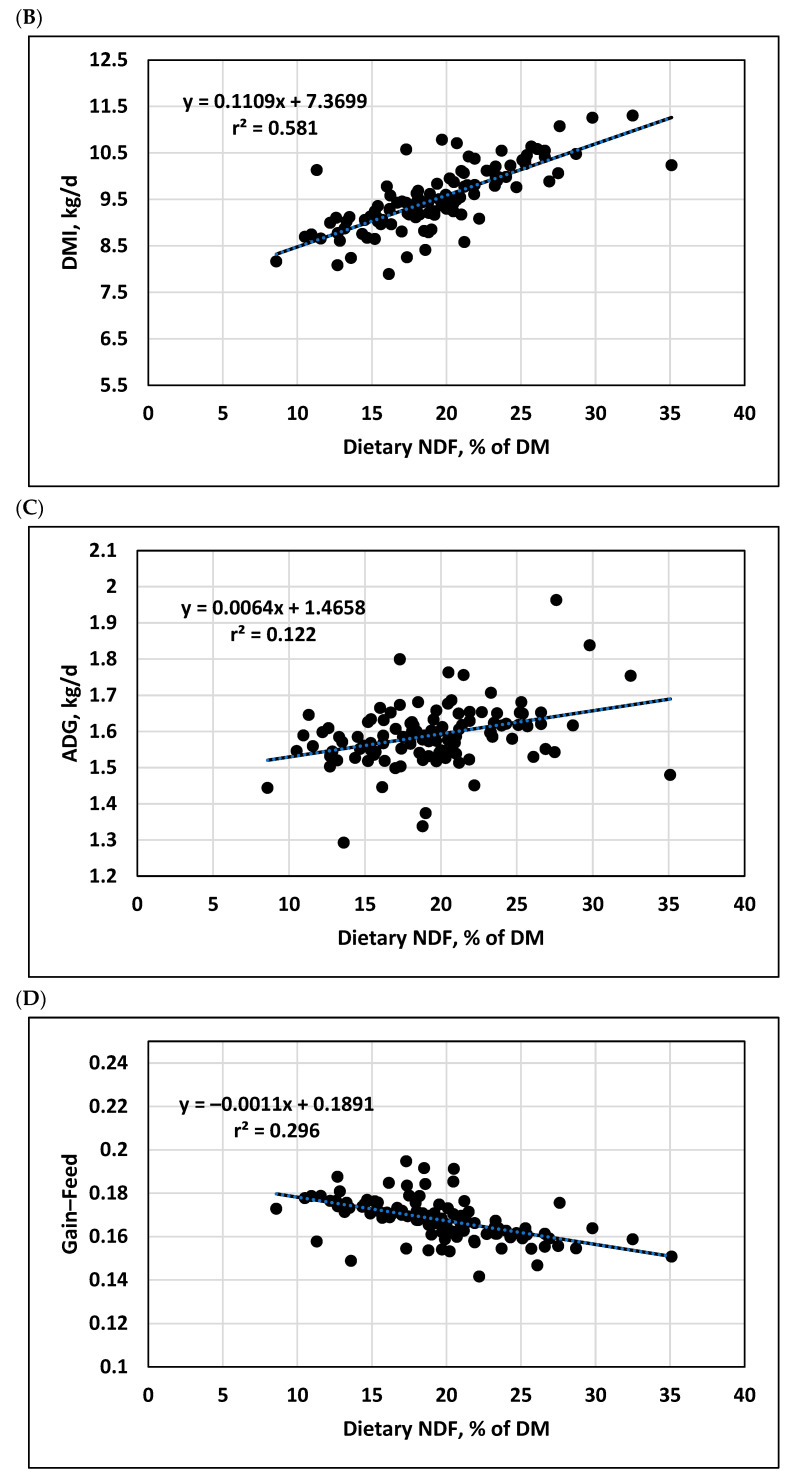

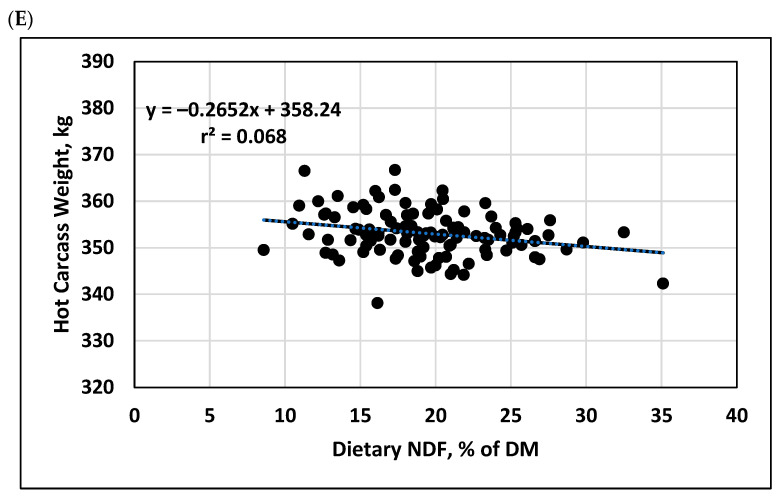
Study-adjusted relationships between growth performance data and dietary concentration of neutral detergent fiber (NDF): (**A**) Dry matter intake (DMI), % of body weight (BW); (**B**) dry matter intake, kg/d; (**C**) average daily gain (ADG), kg/d; (**D**) gain–feed ratio; and (**E**) hot carcass weight, kg. The blue-black dashed line in each figure represents the regression equation.

Regression data for the growth performance variables are shown in Table 2, Table 3, Table 4, Table 5 and Table 6. For each variable, intercept and slope values for the mixed-model regressions are presented, along with measures related to the strength (r^2^, coefficient of determination) and quality (RMSE, root mean square error; CV, coefficient of variation = RMSE divided by the overall mean for the independent variable) of the regression equations using the study-adjusted data. Because regressions for growth performance variables and total dietary NDF showed the largest r^2^ values and least RMSE values among the three measures of fibrousness evaluated, these data are also shown graphically in Figure 1.

### 3.1. Dry Matter Intake

For consistency with previous research [2,5], the DMI regressions were evaluated with DMI expressed both as a percentage of BW (Table 2) and as kilograms/day consumed (Table 3). Among the three independent variables, total dietary NDF was most highly related to DMI (r^2^ = 0.633), with the smallest RMSE, both in terms of absolute value and relative to the mean (i.e., the CV). Between the remaining two variables, the percentage of roughage was more highly related to DMI than the NDF supplied by roughage was, but both variables had much lower r^2^ values than total dietary NDF. Generally, r^2^ values were greater and CV values were less with DMI expressed as a percentage of BW, presumably reflecting the additional variation accounted for by controlling the variable for differences in BW among studies.

**Table 2:** Equations for predicting study-adjusted feedlot dry matter intake as a percentage of body weight from various fiber components developed from a literature database ^1^.

	Regression Coefficients		Regression Statistics ^3^	
Item ^2^	Intercept	Slope	RMSE	r^2^
	Dry matter intake, % of BW			
Dietary roughage, %	1.8956	0.0109	0.0870	0.299
*p*-values ^4^	<0.001	<0.001	CV = 4.38%	
Lower 95% CI	1.8648	0.0077		
Upper 95% CI	1.9264	0.0141		
NDF from roughage, %	1.9145	0.0162	0.0939	0.196
*p*-values ^4^	<0.001	<0.001	CV = 4.73%	
Lower 95% CI	1.8820	0.0099		
Upper 95% CI	1.9470	0.0224		
Dietary NDF, %	1.5517	0.0228	0.0833	0.633
*p*-values ^4^	<0.001	<0.001	CV = 4.17%	
Lower 95% CI	1.4856	0.0195		
Upper 95% CI	1.6178	0.0261		

^1^ Data were adjusted for random intercepts and slopes associated with studies. ^2^ NDF = neutral detergent fiber; CI = confidence interval. ^3^ RMSE = root mean squared error; r^2^ = coefficient of determination; CV = RMSE divided by the overall dependent variable mean, expressed as a percentage. ^4^ Probability that the intercept and slope differ from zero.

**Table 3:** Equations for predicting study-adjusted feedlot dry matter intake (kg/d) from various fiber components developed from a literature database ^1^.

	Regression Coefficients		Regression Statistics ^3^	
Item ^2^	Intercept	Slope	RMSE	r^2^
	Dry matter intake, kg/d			
Dietary roughage, %	9.0729	0.0533	0.4709	0.259
*p*-values ^4^	<0.001	<0.001	CV = 4.95%	
Lower 95% CI	8.9063	0.0361		
Upper 95% CI	9.2396	0.0705		
NDF from roughage, %	9.1560	0.0813	0.4927	0.183
*p*-values ^4^	<0.001	<0.001	CV = 5.18	
Lower 95% CI	8.9853	0.0485		
Upper 95% CI	9.3267	0.1141		
Dietary NDF, %	7.3699	0.1109	0.4529	0.581
*p*-values ^4^	<0.001	<0.001	CV = 4.75%	
Lower 95% CI	7.0102	0.0929		
Upper 95% CI	7.7297	0.1289		

^1^ Data were adjusted for random intercepts and slopes associated with studies. ^2^ NDF = neutral detergent fiber; CI = confidence interval. ^3^ RMSE = root mean squared error; r^2^ = coefficient of determination; CV = RMSE divided by the overall dependent variable mean, expressed as a percentage. ^4^ Probability that the intercept and slope differ from zero.

### 3.2. Average Daily Gain

As with DMI, total dietary NDF concentration was the fiber component most highly related to ADG (Table 4; r^2^ = 0.122 vs. 0.056 and 0.018 for NDF supplied by roughage and dietary roughage, respectively). All slope values were low (average of 0.0043 kg of ADG), and the slope for the percentage of dietary roughage was not significant (*p* = 0.167). Overall, none of the three variables seemed to have important predictive value relative to ADG by feedlot cattle.

**Table 4:** Equations for predicting study-adjusted feedlot average daily gain (kg/d) from various fiber components developed from a literature database ^1^.

	Regression Coefficients		Regression Statistics ^3^	
Item ^2^	Intercept	Slope	RMSE	r^2^
	Average daily gain, kg			
Dietary roughage, %	1.5739	0.0024	0.0926	0.018
*p*-values ^4^	<0.001	0.167	CV = 5.81%	
Lower 95% CI	1.5411	−0.0010		
Upper 95% CI	1.6067	0.0058		
NDF from roughage, %	1.5663	0.0071	0.0827	0.056
*p*-values ^4^	<0.001	0.013	CV = 5.18	
Lower 95% CI	1.5377	0.0016		
Upper 95% CI	1.5950	0.0126		
Dietary NDF, %	1.4658	0.0064	0.0825	0.122
*p*-values ^4^	<0.001	<0.001	CV = 5.19%	
Lower 95% CI	1.4003	0.0031		
Upper 95% CI	1.5313	0.0097		

^1^ Data were adjusted for random intercepts and slopes associated with studies, except for dietary roughage, %, which was adjusted for random intercepts only. ^2^ NDF = neutral detergent fiber; CI = confidence interval. ^3^ RMSE = root mean squared error; r^2^ = coefficient of determination; CV = RMSE divided by the overall dependent variable mean, expressed as a percentage. ^4^ Probability that the intercept and slope differ from zero.

### 3.3. Gain-to-Feed Ratio

The three independent variables were negatively related to G:F (Table 5), with total dietary NDF showing the strongest relationship (r^2^ = 0.296) and the smallest CV, presumably reflecting the stronger relationship between dietary NDF and DMI, one of the two components of G:F. Indeed, the r^2^ values for the percentage of dietary roughage and NDF supplied by roughage were less than one-third the value for total dietary NDF.

**Table 5:** Equations for predicting study-adjusted feedlot gain–feed ratio from various fiber components developed from a literature database ^1^.

	Regression Coefficients		Regression Statistics ^3^	
Item ^2^	Intercept	Slope	RMSE	r^2^
	Gain–feed			
Dietary roughage, %	0.1724	−0.0005	0.0084	0.078
*p*-values ^4^	<0.001	0.003	CV = 4.99%	
Lower 95% CI	0.1694	−0.0008		
Upper 95% CI	0.1754	−0.0002		
NDF from roughage, %	0.1722	−0.0009	0.0084	0.084
*p*-values ^4^	<0.001	0.002	CV = 4.99	
Lower 95% CI	0.1693	−0.0015		
Upper 95% CI	0.1751	−0.0003		
Dietary NDF, %	0.1891	−0.0011	0.0081	0.296
*p*-values ^4^	<0.001	<0.001	CV = 4.81	
Lower 95% CI	0.1827	−0.0014		
Upper 95% CI	0.1955	−0.0008		

^1^ Data were adjusted for random intercepts and slopes associated with studies. ^2^ NDF = neutral detergent fiber; CI = confidence interval. ^3^ RMSE = root mean squared error; r^2^ = coefficient of determination; CV = RMSE divided by the overall dependent variable mean, expressed as a percentage. ^4^ Probability that the intercept and slope differ from zero.

### 3.4. Hot Carcass Weight

None of the three measures of fibrousness were strongly related to HCW (Table 6), with total dietary NDF showing the highest r^2^ value at 0.068. Notably, slopes for the percentage of dietary roughage and NDF supplied by roughage were not significant (*p* ≥ 0.316), with r^2^ values near zero.

**Table 6:** Equations for predicting study-adjusted feedlot hot carcass weight (kg) from various fiber components developed from a literature database ^1^.

	Regression Coefficients		Regression Statistics ^3^	
Item ^2^	Intercept	Slope	RMSE	r^2^
	Hot carcass weight, kg			
Dietary roughage, %	352.16	0.0894	4.8148	0.009
*p*-values ^4^	<0.001	0.316	CV = 1.36%	
Lower 95% CI	350.46	−0.0865		
Upper 95% CI	353.86	0.2653		
NDF from roughage, %	353.23	0.0377	4.8015	0.001
*p*-values ^4^	<0.001	0.816	CV = 1.36%	
Lower 95% CI	351.57	−0.2820		
Upper 95% CI	354.89	0.3575		
Dietary NDF, %	358.24	−0.2652	4.7397	0.068
*p*-values ^4^	<0.001	0.006	CV = 1.34%	
Lower 95% CI	354.47	−0.4533		
Upper 95% CI	362.01	−0.0772		

^1^ Data were adjusted for random intercepts and slopes associated with studies. ^2^ NDF = neutral detergent fiber; CI = confidence interval. ^3^ RMSE = root mean squared error; r^2^ = coefficient of determination; CV = RMSE divided by the overall dependent variable mean, expressed as a percentage. ^4^ Probability that the intercept and slope differ from zero.

## 4. Discussion

### 4.1. Measures of Fiber and Growth Performance

The database used for the current analyses does not include data points used in previous analyses [2,5]. Moreover, unlike the database used in these previous studies, fibrous byproducts were included in the diets in 14 of the 23 studies in the current database. Other than one study that included a cottonseed byproduct, corn gluten feed and distillers grains plus solubles (wet or dry) were the primary sources of digestible fiber used in these studies. Although tabulated in the database, the percentage of fibrous byproduct in the diet and NDF supplied by fibrous byproducts were not included in regression analyses because of either the lack of byproduct in nine studies or the lack of variation in byproduct within the 14 studies that included byproduct NDF. Nonetheless, fibrous byproducts clearly contributed to the total dietary NDF in many studies. Using the same studies as Galyean and Defoor [2], total dietary NDF was found to be highly related (r^2^ > 0.95) to DMI [5], either as a percentage of BW (1.517 + 0.034 × % dietary NDF) or as kilograms/day (6.093 + 0.160 × % dietary NDF), which is consistent with the current results. Indeed, for the DMI as a percentage of BW equation, the intercept reported by Arelovich et al. [5] was within the 95% confidence limits observed in the present study (Table 2). The lower r^2^ value in the present study compared with the earlier work [5] might reflect the lack of byproduct NDF in the database used previously [5], compared with values in the database used for the present analyses.

Galyean and Defoor [2] did not evaluate total dietary NDF in their analyses, but they reported that NDF supplied by roughage accounted for 92% of the variation in study-adjusted DMI as a percentage of BW—a value only slightly less than the follow-up NDF results [5] using the same database. Perhaps the lower r^2^ between DMI and total dietary NDF compared with NDF supplied by roughage noted in the present analyses reflects the inclusion of fibrous byproducts in many of the studies used in the current database. Moreover, the previous authors [2] suggested that roughage sources in feedlot diets could be exchanged on the basis of NDF supplied by roughage. Many of the studies in the current database used this method to balance NDF among roughage sources, which also could have contributed to the lower r^2^ values observed for NDF supplied by roughage noted in the present analyses compared with the previous work.

The results of the current analyses suggest that total dietary NDF concentration is significantly related to DMI by feedlot cattle. Within the range of NDF included in the database (8.6 to 35.1% of DM), each 1% increase in dietary NDF would be expected to increase DMI by 0.023% of BW or 0.11 kg/d. In addition, the relationship is linear within this range of NDF concentrations, as the quadratic effect of NDF concentration was not significant for either DMI expressed as a percentage of BW or as kilograms per day (*p* > 0.99 and *p* > 0.45, respectively, for study-adjusted values). Although exchanging roughages based on NDF supplied by roughage as suggested previously [2] has proved to be a practical approach to maintain DMI in feedlot diets, present data suggest that total dietary NDF might be a more useful measure for exchanging dietary ingredients, especially when diets contain significant proportions of fibrous byproducts. Thus, when ingredient sources are changed in feedlot diets, particularly roughages and fibrous byproducts that are significant sources of NDF, balancing diets to a specific NDF concentration would be expected to maintain a relatively constant DMI.

In the present analysis, ADG was not strongly related to any of the three measures of dietary fiber. This result suggests that feedlot ADG remains relatively constant with changes in roughage concentration, NDF supplied by roughage, and total dietary NDF concentration, at least within the ranges for these values in the data set. Growth performance measures besides DMI were not evaluated in the previous analyses [2,5], although other published research related to the effects of roughage concentration in feedlot diets might provide insights into expected relationships. For example, alfalfa hay concentrations of 2, 6, 10, and 14% of the DM were compared in feedlot diets that contained 25% (DM basis) wet corn distillers grains plus solubles [8]. For the overall feeding period, ADG responded quadratically (*p* = 0.01), with a lesser ADG noted for the 14% alfalfa hay diet. Similarly, roughage concentrations from alfalfa hay at 7.5, 10, or 12.5% of the DM were compared in diets that had either 15 or 30% wet distillers grains plus solubles [9]. Overall, ADG was not affected by roughage concentration (*p* > 0.10), and roughage concentration did not interact with distillers grain concentration.

The correlation between dietary NEm and NEg with total dietary NDF in the current database was negative (r = −0.33 and −0.35, respectively; *p* < 0.001), but it seems that feedlot cattle compensate for this fiber-related energy dilution by increasing DMI, thereby maintaining ADG, at least up to a point. In the current database, the ADG slope for total dietary NDF was positive, although the effect was very small (0.0064 kg of ADG per unit increase in NDF). This finding might indicate that the positive effects of dietary fiber in feedlot diets (e.g., increased salivation and decreased gastrointestinal tract acidity [3]) could result in increasing DMI beyond the point of compensation, thereby increasing intake of energy above maintenance, with an associated increase in ADG. Nonetheless, given the low r^2^ for the relationships between ADG and the three measures of dietary fibrousness evaluated in the present study, this suggestion is speculative at best.

The negative relationship in the present database between G:F and measures of dietary fiber reflects the increase in DMI and relatively stable ADG associated with increased dietary NDF discussed previously. The result of these associations was a reasonably strong relationship between G:F and total dietary NDF concentration that is consistent with previous research [8,9]. In contrast, the lack of a relationship between HCW and measures of dietary fibrousness suggests that HCW is more reflective of ADG than other growth performance measures, which is supported by the correlation between these two variables in the current database (r = 0.63; *p* < 0.001).

### 4.2. Physically Effective Fiber

Physically effective fiber is an important concept in dairy nutrition [10]. The concept recognizes that the particle size of fiber is associated with chewing and rumination, both of which affect salivary flow and ruminal buffering. Mertens [11] defined physically effective NDF (peNDF) as the fraction of the diet retained on a 1.18 mm sieve (vertical sieving of the dry diet) multiplied by the total NDF of the diet, ultimately recommending 20 to 22% peNDF in diets for lactating cows to maintain milk fat at 3.4% and an average ruminal pH of 6.0, respectively. Subsequent research has involved sieving as-fed diets using various screens, with the suggestion [10] that the Penn State Particle Separator with a 4 mm sieve might provide values comparable to the original dry sieving method proposed by Mertens [11]. Grant [10] detailed the assumptions associated with calculations of peNDF, which include uniform distribution of NDF, uniform chewing activity for all particles > 1.18 mm, and a lack of differences in fragility among sources of NDF.

Whether peNDF is an important measurement for beef feedlot diets remains an open question. Diets for lactating dairy cows typically contain three to five times as much traditional roughage as feedlot diets, so the effects of peNDF could be muted in feedlot diets because of the lower concentrations of coarse roughage.

Several recent studies have evaluated peNDF in feedlot diets, primarily with a focus on changes in ruminal pH and rumination time. Gentry et al. [12] compared steam-flaked corn-based diets with 5 or 10% corn stalks (peNDF value of 50.5%) vs. a diet with 5% corn stalks with a peNDF of 91.9% (peNDF values determined by sieving with a 4 mm screen). Diets also contained 24.4 to 30% wet corn gluten feed. Daily DMI was greatest with the 5% high-peNDF corn stalk diet and least with the 10% low-peNDF corn stalk diet, but ADG and G:F did not differ among treatments. Rumination time (minutes per day) was greatest with the 10% low-peNDF corn stalk diet and least with the 5% low-peNDF corn stalk diet. Using beef heifers [13], diets (a barley–corn mixture as the grain source) with 15% barley straw that varied in peNDF were compared. Ultimate dietary peNDF concentrations (based on a 4 mm sieve) were 6.4, 10.4, 13.6, and 15.4% of DM. Dry matter and NDF intakes decreased linearly with increasing peNDF concentration. Rumination time also increased linearly, and the time below ruminal pH thresholds ranging from 5.5 to 5.8 decreased linearly as dietary peNDF increased. Heifers in the two highest peNDF diets sorted against straw particles > 4 mm. Feeding barley-based diets to beef heifers [14], it was reported that increasing peNDF (8- or 1.18 mm sieves) supplied by barley silage (0, 4, 8, or 14% barley silage) increased the minimum and average ruminal pH and quadratically affected time under pH thresholds of 5.2, 5.5, and 5.8. Rumination time (minutes per day) increased quadratically with increasing barley silage. The effects of peNDF supplied by sugarcane were evaluated in Nellore bulls fed whole-corn-based diets [15]. Intakes of total digestible nutrients, DM, and organic matter responded quadratically to peNDF, with the greatest responses between 6.14 and 10.2% dietary peNDF (based on a 4 mm sieve). Likewise, ruminal pH increased linearly with increasing peNDF in the diet. Pereira et al. [16] compared barley and wheat silage with high or low-peNDF fed at 10% of the dietary DM fed to crossbred steers for 123 d. A fifth treatment of a low-peNDF diet with added wheat straw (total of 10% roughage) was used to assess the effects of adding undigestible NDF to the diet. Treatments did not affect DMI, ADG, or G:F, but the cattle fed low-peNDF wheat silage had a greater incidence of severe liver abscesses than cattle in other treatment groups. In steam-flaked corn-based diets containing 8% corn stalks [17], treatments included wet distillers grains plus solubles (20% of DM), wet corn gluten feed (20% of DM), or a combination of wet distillers (10% of DM) and wet corn gluten feed (20% of DM), along with a control diet that did not contain fibrous byproducts. The peNDF of diets was determined by sieving through either 4 or 8 mm screens. Results suggested that both wet distillers grains and wet corn gluten feed positively affected ruminal pH compared with the non-byproduct control diet, and the authors concluded that dietary starch concentrations might influence ruminal pH to a greater extent than the physical nature of the diet.

Overall, results of the recent research on peNDF in feedlot diets suggest positive relationships between peNDF and ruminal pH and rumination time, but the relationship between peNDF concentration in the diet and feedlot growth performance is less clear. Moreover, the concentration of peNDF needed to achieve positive ruminal effects has not been established. Challenges with the available research on the effects of peNDF on feedlot cattle include different sieve sizes and methods (as-fed or dry) to define peNDF, and the fact that in some of the studies, total dietary NDF increased along with peNDF, making it unclear whether peNDF is a more effective measure than total dietary NDF alone. In addition, studies vary considerably in the inclusion of digestible byproduct fiber sources, which seem to have some degree of physical effectiveness, or at least the inclusion of these ingredients decreases total dietary starch content, thereby lessening the negative effects of starch fermentation on ruminal pH.

Although some of the studies included in the current database evaluated physically effective fiber, most did not. Thus, the current database cannot be used to assess the potential effects of peNDF on the growth performance of feedlot cattle. There is a need for research to evaluate the relationship between both peNDF and total dietary NDF in feedlot diets and measures of ruminal fermentation, particularly changes in ruminal pH and rumination time, as well as production outcomes related to diet acidity, like liver abscesses.

## 5. Summary and Conclusions

Based on analysis of the literature data, the total NDF concentration of beef feedlot diets is significantly related to DMI, both as a percentage of BW and as the total amount consumed per day. In addition, total dietary NDF accounted for more variation in DMI than either the percentage of dietary roughage or the percentage of NDF supplied by roughage. Dietary NDF also accounted for more variation in ADG, G:F, and HCW than the percentage of roughage and the percentage of NDF supplied by roughage did, but relationships between these growth performance variables and dietary fiber components were not as strong as the relationships with DMI. Although additional research is needed on the role of physically effective fiber in feedlot growth performance, present results suggest that total dietary NDF concentration should be an effective tool for exchanging dietary ingredients to achieve similar growth performance responses, particularly DMI.

## Figures and Tables

**Table 1:** Descriptive statistics for the literature database used to evaluate the effects of measures of fibrousness in feedlot diets.

Variable ^1^	Mean	SD	Minimum	Maximum
	Across-Study Values			
No. of observations	110	-	-	-
Initial BW, kg	369.1	45.56	310.5	493
Final BW, kg	585.8	52.61	474	733
Hot carcass weight, kg	357.4	40.33	260.7	425
Days on feed	131.2	27.77	70	175
DMI, % of average BW	1.99	0.22	1.36	2.58
DMI, kg/d	9.51	1.39	6.5	11.8
ADG, kg/d	1.63	0.26	0.77	2.31
G:F	0.17	0.02	0.12	0.23
Dietary roughage, %	8.2	5.20	0	30.0
NDF in roughage, %	51.8	17.72	0	85.9
NDF from roughage, %	4.4	2.85	0	15.2
Dietary fibrous byproduct, %	13.6	14.53	0	40.1
NDF in byproduct, %	18.9	16.4	0	54.0
NDF from byproduct, %	4.2	4.42	0	12.4
Dietary NEm, Mcal/kg	2.10	0.14	1.27	2.31
Dietary NEg, Mcal/kg	1.43	0.11	1.22	1.86
Dietary NDF, %	19.5	4.79	8.58	35.1
Dietary starch, %	51.5	8.58	31.0	67.5
Dietary CP, %	14.5	1.83	11.7	21.2
	Within-Study Ranges			
Dietary roughage, %	9.4	5.37	0.0	22.5
Dietary fibrous byproduct, %	10.1	14.60	0.0	40.1
Dietary NDF, %	5.2	3.12	1.1	12.4
NDF from roughage, %	4.9	2.88	1.4	11.4
NDF from byproduct, %	3.3	4.72	0.0	12.4

^1^ SD = standard deviation; BW = body weight; DMI = dry matter intake; ADG = average daily gain; G:F = gain-to-feed ratio; NDF = neutral detergent fiber; CP = crude protein. All dietary values are expressed as a percentage of the dry matter.

## Data Availability

The literature database is available for download at: https://www.depts.ttu.edu/afs/burnett_center/feedlot-fiber-database.php, accessed on 6 November 2025.

## Appendix A

### Articles Used in the Database

Benton, J.R.; Watson, A.K.; Erickson, G.E.; Klopfenstein, T.J.; Vander Pol, K.J.; Meyer, N.F.; Greenquist, M.A. Effects of roughage source and inclusion in beef finishing diets containing corn wet distillers’ grains plus solubles. *J. Anim Sci.* **2015**, *93*, 4358–4367. https://doi.org/10.2527/jas.2015-9211.

Contadini, M.A.; Ferreira, F.A.; Corte, R.R.S.; Antonelo, D.S.; Gómez, J.F.M.; Silva, S.L. Roughage levels impact on performance and carcass traits of finishing Nellore cattle fed whole corn grain diets. *Trop. Anim. Health Prod.* **2017**, *49*, 1709–1713. https://doi.org/10.1007/s11250-017-1381-x.

de Melo, A.H.F.; Marques, R.S.; Gouvêa, V.N.; de Souza, J.; Batalha, C.D.A.; Basto, D.C.; Millen, D.D.; Drouillard, J.S.; Santos, F.A.P. Effects of dietary roughage neutral detergent fiber levels and flint corn processing method on growth performance, carcass characteristics, feeding behavior, and rumen morphometrics of *Bos indicus* cattle. *J. Anim. Sci.* **2019**, *97*, 3562–3577. https://doi.org/10.1093/jas/skz197.

Farran, T.B.; Erickson, G.E.; Klopfenstein, T.J.; Macken, C.N.; Lindquist, R.U. Wet corn gluten feed and alfalfa hay levels in dry-rolled corn finishing diets: Effects on finishing performance and feedlot nitrogen mass balance. *J. Anim. Sci.* **2006**, *84*, 1205–1214. https://doi.org/10.2527/2006.8451205x.

Gentry, W.W.; Weiss, C.P.; Meredith, C.M.; McCollum, F.T.; Cole, N.A.; Jennings, J.S. Effects of roughage inclusion and particle size on performance and rumination behavior of finishing beef steers. *J. Anim. Sci.* **2016**, *94*, 4759–4770. https://doi.org/10.2527/jas.2016-0734.

Gorocica-Buenfil, M.A.; Loerch, S.C. Effect of cattle age, forage level, and corn processing on diet digestibility and feedlot performance. *J. Anim. Sci.* **2005**, *83*, 705–714. https://doi.org/10.2527/2005.833705x.

Hales, K.E.; McMeniman, J.P.; Leibovich, J.; Vasconcelos, J.T.; Quinn, M.J.; May, M.L.; DiLorenzo, N.; Smith, D.R.; Galyean, M.L. Effects of varying bulk densities of steam-flaked corn and dietary roughage concentration on in vitro fermentation, performance, carcass quality, and acid-base balance measurements in finishing steers. *J. Anim. Sci.* **2010**, *88*, 1135–1147. https://doi.org/10.2527/jas.2009-2400.

Hales, K.E.; Freetly, H.C.; Shackelford, S.D.; King, D.A. Effects of roughage concentration in dry-rolled corn-based diets containing wet distillers grains with solubles on performance and carcass characteristics of finishing beef steers, *J. Anim. Sci.* **2013**, *91*, 3315–3321. https://doi.org/10.2527/jas.2012-5942.

Koenig, K.M.; Beauchemin, K.A. Optimum extent of barley grain processing and barley silage proportion in feedlot cattle diets: Growth, feed efficiency and fecal characteristics. *Can. J. Anim. Sci.* **2011**, *91*, 411–422. https://doi.org/10.4141/CJAS2010-039.

Koenig, K.M.; Chibisa, G.E.; Penner, G.B.; Beauchemin, K.A. Optimum roughage proportion in barley-based feedlot cattle diets: growth performance, feeding behavior, and carcass traits. *J. Anim. Sci.* **2020**, *98*, skaa299. https://doi.org/10.1093/jas/skaa299.

Marques, R.S.; Chagas, L.J.; Owens, F.N.; Santos, F.A.P. Effects of various roughage levels with whole flint corn grain on performance of finishing cattle. *J. Anim. Sci.* **2016**, *94*, 339–348. https://doi.org/10.2527/jas.2015-9758.

May, M.L.; Quinn, M.J.; DiLorenzo, N.; Smith, D.R.; Galyean, M.L. Effects of roughage concentration in steam-flaked corn-based diets containing wet distillers grains with solubles on feedlot cattle performance, carcass characteristics, and in vitro fermentation. *J. Anim. Sci.* **2011**, *89*, 549–559. https://doi.org/10.2527/jas.2010-3049.

McDaniel, Z.S.; Galyean, M.L.; Broadway, P.R.; Carroll, J.A.; Burdick Sanchez, N.C.; Hanratty, A.N.; Dornbach, C.W.; Line, D.J.; Smock, T.M.; Manahan, J.L.; et al. Effects of increasing the concentration of neutral detergent fiber in roughage and bulk density of steam-flaked corn on growth performance, carcass characteristics, and liver abscesses of finishing beef steers fed diets without tylosin phosphate. *Appl. Anim. Sci.* **2024**, *40*, 269–278. https://doi.org/10.15232/aas.2023-02484.

Nelson, M.L.; Marks, D.J.; Busboom, J.R.; Cronrath, J.D.; Falen, L. Effects of supplemental fat on growth performance and quality of beef from steers fed barley-potato product finishing diets: I. Feedlot performance, carcass traits, appearance, water binding, retail storage, and palatability attributes. *J. Anim. Sci.* **2004**, *82*, 3600–3610. https://doi.org/10.2527/2004.82123600x.

Niwa, M.V.G.; Ítavo, L.C.V.; Ítavo, C.C.B.F.; Gomes, M.N.B.; Longhini, V.Z.; Cunha, C.S.; Dias, A.M.; Difante, G.S.; Vedovatto, M.; Gomes, R.C.; et al. Fiber sources on performance and carcass and meat characteristics of feedlot Nellore young bulls. *Anim. Physiol. Anim. Nutr.* **2025**, *109*, 551–559. https://doi.org/10.1111/jpn.14072.

Parsons, C.H.; Vasconcelos, J.T.; Swingle, R.S.; Defoor, P.J.; Nunnery, G.A.; Salyer, G.B.; Galyean, M.L. Effects of wet corn gluten feed and roughage levels on performance, carcass characteristics, and feeding behavior of feedlot cattle. *J. Anim. Sci.* **2007**, *85*, 3079–3089. https://doi.org/10.2527/jas.2007-0149.

Quinn, M.J.; May, M.L.; DiLorenzo, N.; Ponce, C.H.; Smith, D.R.; Parr, S.L.; Galyean, M.L. Effects of roughage source and distillers grain concentration on beef cattle finishing performance, carcass characteristics, and in vitro fermentation. *J. Anim. Sci.* **2011**, *89*, 2361–2642. https://doi.org/10.2527/jas.2010-3563.

Swanson, K.C.; Carlson, Z.E.; Ruch, M.C.; Gilbery, T.C.; Underdahl, S.R.; Keomanivong, F.E.; Bauer, M.L.; Islas, A. Influence of forage source and forage inclusion level on growth performance, feeding behavior, and carcass characteristics in finishing steers. *J. Anim. Sci.* **2017**, *95*, 1325–1334. https://doi.org/10.2527/jas2016.1157.

Terry, S.A.; Yang, W.; Beauchemin, K.A.; Schwartzkopf-Genswein, K.S.; Penner, G.B.; Wood, K.M.; McAllister, T.A. Effect of undigestible neutral detergent fiber concentration in finishing diets containing dry-rolled or steam-rolled barley for feedlot steers. *J. Anim. Sci.* **2025**, *103*, skae392. https://doi.org/10.1093/jas/skae392.

Toseti, L.B.; Goulart, R.S.; Gouvêa, V.N.; Acedo, T.S.; Vasconcellos, G.S.F.M.; Pires, A.V.; Leme, P.R.; Netto, A.S.; Silva, S.L. Effects of a blend of essential oils and exogenous α-amylase in diets containing different roughage sources for finishing beef cattle. *Anim. Feed Sci. Technol.* **2020**, *269*, 114643. https://doi.org/10.1016/j.anifeedsci.2020.114643.

Uwituze, S.; Parsons, G.L.; Shelor, M.K.; Depenbusch, B.E.; Karges, K.K.; Gibson, M.L.; Reinhardt, C.D.; Higgins, J.J.; Drouillard, J.S. Evaluation of dried distillers grains and roughage source in steam-flaked corn finishing diets. *J. Anim. Sci.* **2010**, *88*, 258–274. https://doi.org/10.2527/jas.2008-1342.

Word, A.B.; Karr, K.J.; Holland, B.P.; Maxwell, C.L.; Linneen, S.K.; Defoor, P.J. Removing tylosin phosphate from finishing diets with increasing roughage concentrations affects growth performance, carcass characteristics, and prevalence of liver abscesses of finishing steers. *Appl. Anim. Sci.* **2024**, *40*, 260–268.https://doi.org/10.15232/aas.2023-02489.

Yang, W.Z.; Li, Y.L.; McAllister, T.A.; McKinnon, J.J.; Beauchemin, K.A. Wheat distillers grains in feedlot cattle diets: Feeding behavior, growth performance, carcass characteristics, and blood metabolites. *J. Anim. Sci.* **2012**, *90*, 1301–1310. https://doi.org/10.2527/jas.2011-4372.
